# Predictors of response and anti-TNF drugs comparative efficacy

**DOI:** 10.1186/1479-5876-9-S2-P55

**Published:** 2011-11-23

**Authors:** Helena Canhão, Ana F Mourão, Fernando Martins, Maria J Santos, Canas Silva, Joaquim Pereira, JA Pereira Silva, José Costa, Domingos Araujo, Candida Silva, Eugenia Simões, Catia Duarte, José Antonio P Silva, Fernando Pimentel, Jaime Branco, João E Fonseca

**Affiliations:** 1 Rheumatology Research Unit, Instituto de Medicina Molecular, Lisbon, Portugal

## Background

Tumor necrosis factor (TNF) antagonist therapies changed the rheumatoid arthritis treatment paradigm but arguments to choose among anti-TNF are lacking.

The aim of this work was to evaluate the comparative effectiveness of adalimumab, etanercept and infliximab over 1 year in clinical practice.

Secondary aims were to look for clinical predictors of response.

## Methods

Analyses were performed upon Reuma.pt. Response to therapy was defined according to EULAR criteria. Probability of response was modeled. Multivariate logistic regression model predicting response over 1 year with all variables and automated stepwise selection models were built. In addition, we performed analyses using propensity score 1:1:1 nearest neighbor matching algorithms to obtain comparable groups regarding baseline features.

## Results

632 RA patients, 250 treated with etanercept, 206 with infliximab and 161 with adalimumab were evaluated.

At baseline visit, infliximab treated patients were significantly older, had lower education level, more comorbidities and more concomitant therapies.

We have found significant negative association with anti-TNF response and smoking, presence of RF and ACPA and higher physician VAS at baseline.

In unadjusted analysis, proportion of good response over 1 year was 59.2% in etanercept, 52% in infliximab and 59.6% in adalimumab group (p=0.21). GLM showed no significant differences between the 3. Multivariate logistic regression analyses demonstrated similar effectiveness. Also, no significant differences in treatment response were detected after applying the matching propensity score analysis.

## Conclusion

Smoking, RF, ACPA and physician VAS were identified as predictors of response. In our study using different statistic techniques, we observed similar efficacy over 1 year for the 3 anti-TNF agents studied.

